# Modulatory effect of sesamol on DOCA-salt-induced oxidative stress in uninephrectomized hypertensive rats

**DOI:** 10.1007/s11010-013-1647-1

**Published:** 2013-04-11

**Authors:** Govindasamy Hemalatha, Kodukkur Viswanathan Pugalendi, Ramalingam Saravanan

**Affiliations:** Department of Biochemistry and Biotechnology, Faculty of Science, Annamalai University, Annamalainagar, 608 002 Tamilnadu India

**Keywords:** Hypertension, Uninephrectomy, DOCA-salt, Sesamol, Oxidative stress

## Abstract

The present study was undertaken to investigate the antihypertensive and antioxidant effects of sesamol on uninephrectomized deoxycorticosterone acetate (DOCA)-salt-induced hypertensive rats. Hypertension was induced in surgically single-kidney-removed (left) adult male albino Wistar rats, weighing 180–200 g, by injecting DOCA (25 mg/kg BW) subcutaneously twice a week for 6 weeks, with saline instead of tap water for drinking. Rats were treated with three different doses of sesamol (50, 100 and 200 mg/kg BW) post-orally by gavage daily for 6 weeks. Hypertension was revealed by increased systolic and diastolic blood pressure and the toxicity of DOCA-salt was determined using hepatic marker enzymes, aspartate aminotransferase, alanine aminotransferase, alkaline phospatase and gamma-glutamyl transpeptidase; and, lipid peroxidative markers, thiobarbituric acid reactive substances, lipid hydroperoxides and conjugated dienes were assayed. The activities of enzymatic antioxidants, superoxide dismutase, catalase and glutathione peroxidase and the levels of non-enzymatic antioxidants (vitamin C, vitamin E and reduced glutathione) were evaluated in erythrocytes, plasma and tissues. Post-oral administration of sesamol at the dosage of 50 mg/kg BW remarkably decreased systolic and diastolic blood pressure, hepatic marker enzyme activities and lipid peroxidation products and also enhanced the antioxidant activity. The biochemical observations were also supported by histopathological examinations of the rat liver, kidney and heart sections. These results suggest that sesamol possesses antihypertensive and antioxidant effects.

## Introduction

Hypertension is the most common cardiovascular disease, which affects approximately 26 % of the adult population worldwide [1], and its prevalence is predicted to increase by 60 % by 2025. Elevated blood pressure is one of the most important and easily remediable factors for adverse cardiovascular outcomes, including myocardial infarction, renal failure and death. Hypertension could be induced in specified experimental models such as deoxycorticosterone acetate-sodium chloride (DOCA-salt) rats. The prolonged administration of the synthetic mineralocorticoid, DOCA, with salt promotes increased concentrations of aldosterone, which results in sodium retention in the distal nephron of the kidney and creates a distinguished volume-dependent hypertension in uninephrectomized (UNX) rats [2]. In DOCA-salt hypertension, there is a marked elevation of renal vascular resistance and a decrease in renal blood flow, and these abnormalities in renal hemodynamics are thought to be involved in the development and maintenance of hypertension [3].

In biological systems, about 1–2 % of inhaled O_2_ may be converted to superoxide anion, and its dismutation gives rise to H_2_O_2_. Free radical concentration is maintained stable by controlling production rates and by the scavenging capacity of the antioxidant enzymes. When this equilibrium is broken, reactive oxygen species (ROS), in excess of normal requirements of the cells, may randomly damage the final structural and functional integrity due to their high chemical reactivity. They may either by directly modifying cellular DNA, proteins and lipids or by initiating chain reactions bring about extensive oxidative damage to these critical molecules [4].

**Fig. 1:**
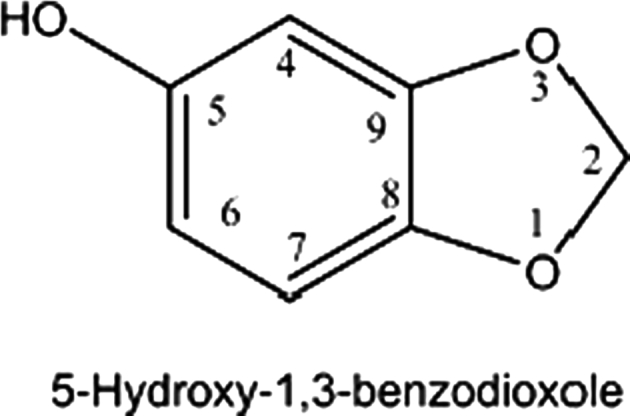
Structure of sesamol

Sesame products, from *Sesamum* *indicum*, have been known as traditional health foods and have been used in ancient medicine for a long time. Previous studies from our laboratory revealed that sesame oil possesses antihypertensive activity in humans [5]. Lignans are an important class of plant-derived compounds known to possess a variety of biological activities such as antimitotic, antiviral and antiatherosclerotic activities [6]. They provide protection against several ROS, the development of hypertension and other oxidative stress-mediated dysfunctions. Sesamol (5-hydroxy-1,3-benzodioxole; Fig. 1), a phytonutrient of the class of lignans, is a lipophilic compound present in sesame and is known for its antioxidant role [7]. The biological effects of sesamol on health have been determined as follows: Sesamol exhibited powerful inhibitory effects on lipid peroxidation [8] and carried out the synergistic suppression of carcinogenesis when combined with other antioxidants. Sesamol, a metabolic regulator, has also been reported to act as an anti-ageing [9], antihepatotoxic [10] agent and also inhibits atherosclerosis [11]. Vennila et al. [12] stated that sesamol has cardioprotective activities in myocardial infarcted rats.

An extensive literature survey has shown that no work has been done for its antihypertensive property. It is of great interest to evaluate whether administration of sesamol attenuates the blood pressure and oxidative stress in DOCA-salt hypertension.

## Materials and methods

### Animals

Albino Wistar male rats (*Rattus norvegicus*), 11–13 weeks old and weighing 180–200 g, were obtained from the Central Animal House, the Department of Experimental Medicine, Rajah Muthiah Medical College and Hospital, Annamalai University, India, and were housed in the central animal house with 12-h light and 12-h dark cycles. The animals were randomized into experimental and control groups and housed six in a polypropylene cage. The control and experimental animals were provided food and water ad libitum. The whole experiment was carried out according to the guidelines of the Committee for the Purpose of Control and Supervision of Experiments on Animals, New Delhi, India, and approved by the Animal Ethics Committee of Annamalai University (Reg No: 160/1999/CPCSEA).

### Chemicals

Sesamol, DOCA and dimethyl formamide (DMF) were purchased from Sigma-Aldrich Chemical Company, St. Louis, MO. All other chemicals used in this study were of analytical grade, obtained from Sisco Research Laboratories or Himedia, Mumbai, India.

### Method of uninephrectomy

Rats were anaesthetized by an intraperitoneal injection of ketamine (75 mg/kg BW). The skin above the left kidney was shaved, cleaned and applied with iodine-based antiseptic. The kidney was visualized by a left lateral abdominal incision (1 cm long) and freed from the surrounding tissues and pulled out gently. The left renal artery and ureter were tied by a silk thread, and then the left kidney was removed and weighed. The muscle and skin layers were closed separately using a chromic sterile absorbable suture. The animals were allowed to recover for 1 week.

### Induction of hypertension

After the recovery period, UNX animals were given twice weekly subcutaneous injections of DOCA-salt (25 mg/kg BW) in 0.4 mL of DMF (vehicle) solution and salt was administered by substitution of 1 % NaCl solution for drinking water ad libitum throughout the experimental period.

### Experimental protocol

The rats were randomly divided into six groups each consisting of six rats: group 1—UNX rats; group 2—UNX rats treated with sesamol alone (200 mg/kg BW); group 3—DOCA-salt hypertensive rats; groups 4, 5 and 6 were hypertensive rats which received different doses of sesamol 50, 100 and 200 mg/kg BW, respectively. Sesamol was freshly solubilised in water and administered post-orally through a gavage once a day for 6 weeks between 9:00 and 10:00 am.

1. UNX-control rats
2. UNX-control rats + sesamol (200 mg/kg BW)
3. DOCA-salt
4. DOCA-salt + sesamol (50 mg/kg BW)
5. DOCA-salt + sesamol (100 mg/kg BW)
6. DOCA-salt + sesamol (200 mg/kg BW)

After the experimental period, all the animals were anaesthetized by an intramuscular injection of ketamine and sacrificed by cervical dislocation and biochemical studies were conducted on the plasma, erythrocytes, liver, kidney and heart samples of the control and experimental animals.

### Blood pressure measurement

Systolic and diastolic blood pressure was measured and documented every week during the experimental period by the tail-cuff method (IITC, model 31, Woodland Hills, CA, USA). The animals were placed in a heated chamber at an ambient temperature of 30–34 °C for 15 min; from each animal, 1–9 blood pressure values were recorded. The lowest three readings were averaged to obtain a mean blood pressure. All recordings and data analyses were done using a computerized data acquisition system and software.

### Biochemical estimations

The activities of aspartate aminotransferase (AST) and alanine aminotransferase (ALT) were assayed by the method of Reitman and Frankel [13]. Alkaline phospatase (ALP) and gamma-glutamyl transpeptidase (GGT) were assessed by the methods of Kind and King [14] and Rosalki and Rau [15], respectively. Thiobarbutric acid reactive substances (TBARS), lipid hydroperoxides (LOOH) and conjugated dienes (CD) were estimated by the methods of Niehaus and Samuelson [16], Jiang et al. [17] and Rao and Recknagel [18], respectively. The non-enzymatic antioxidants GSH, vitamin C and vitamin E were estimated by the methods of Ellman [19], Roe and Kuether [20] and Baker et al. [21], respectively. The activities of SOD, CAT and GPx were measured by the methods of Kakkar et al. [22], Sinha [23] and Rotruck et al. [24], respectively.

### Histopathological examination of hepatic, renal and cardiac tissues

The liver, kidney and heart tissues obtained from all experimental groups were washed immediately with 0.9 % saline and then fixed in 10 % buffered neutral formalin. After fixation, the tissues were embedded in paraffin wax. Then, the tissues were sectioned (5–6 μm) and stained with haematoxylin and eosin (H&E) dye and examined under a high power microscope (Nikon ECLIPSE TS 100; Japan) and photomicrographs were taken.

### Statistical analysis

The data are expressed as mean ± S.D. Statistical comparisons were performed by one-way analysis of variance (ANOVA) followed by Duncan’s multiple range test (DMRT) using statistical package for the social science (SPSS) software version 11.5. The results were considered statistically significant if the *p*-values were 0.05 or less.

## Results

**Fig. 2:**
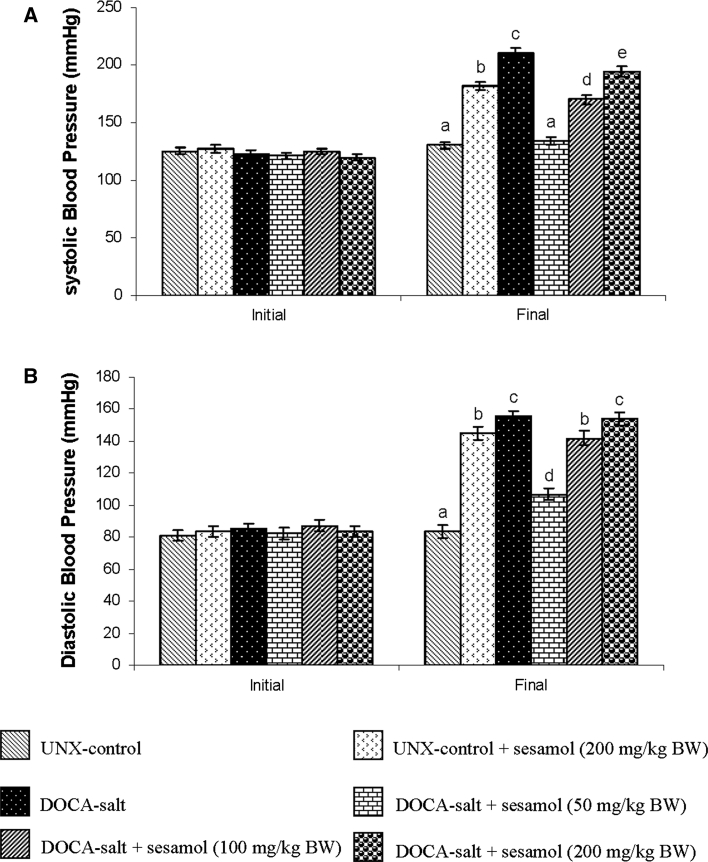
**a** and **b** Effect of sesamol on the systolic and diastolic blood pressure in UNX-control and DOCA-salt hypertensive rats. Values are mean ± SD for six rats. Values not sharing a common superscript differ significantly at *p* < 0.05 (DMRT)

**Fig. 3:**
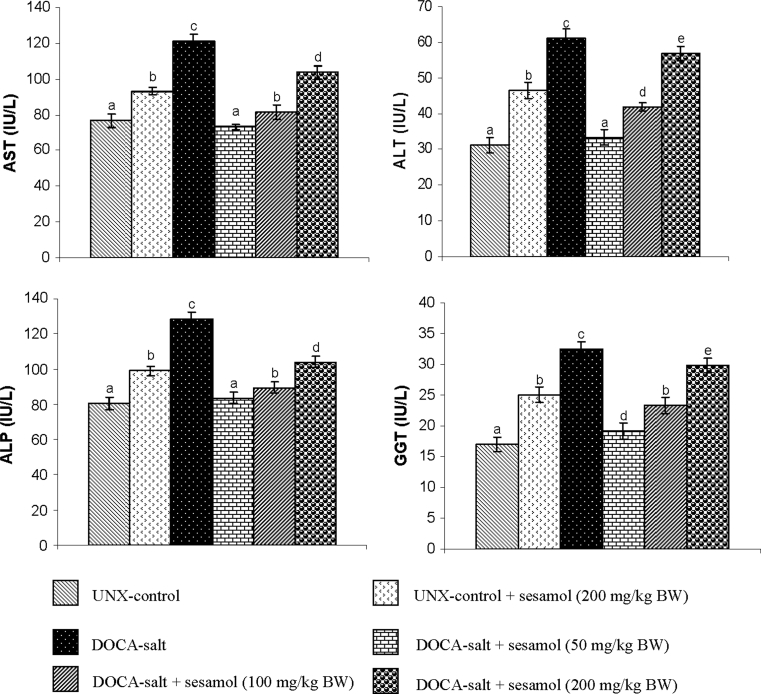
Effect of sesamol on the hepatic marker enzymes of serum in UNX-control and DOCA-salt hypertensive rats. Values are mean ± SD for six rats. Values not sharing a common superscript differ significantly at *p* < 0.05 (DMRT)

Figure 2a, b summarizes the effect of sesamol on systolic and diastolic blood pressure of DOCA-treated and UNX-control rats, respectively. The systolic and diastolic blood pressure of DOCA-salt-treated hypertensive rats was significantly higher than the UNX-control; administration of sesamol at three different doses (50, 100 and 200 mg/kg BW) to the hypertensive rats produced significant lowering effects on the blood pressure and 50 mg/kg BW dosage was better than the other two doses. To study the toxic effect of sesamol, the activities of serum hepatic marker enzymes (AST, ALT, ALP and GGT) in the control and experimental animals are assayed and given in Fig. 3. The activities of hepatic marker enzymes increased in DOCA-salt-induced rats and treatment with sesamol significantly restored the activities of the marker enzymes.

Table 1 depicts the levels of TBARS, LOOH and CD in the plasma, liver, kidney and heart of the UNX-control and experimental animals in each group. The levels of lipid peroxidative markers increased significantly in DOCA-salt-induced hypertensive rats and treatment with sesamol resulted in a significant decrease in the levels. Table 2 reveals the effect of sesamol on the levels of plasma and tissue non-enzymatic antioxidants such as vitamin C, vitamin E and GSH in UNX-control and DOCA-salt hypertensive rats. The levels of Vit C, Vit E and GSH diminished significantly in hypertensive rats and treatment with sesamol increased significantly the non-enzymatic antioxidants to near-normal levels. Table 3 shows the effect of sesamol on enzymatic antioxidant activities in the erythrocytes, liver, kidney and heart of UNX-control and DOCA-salt-induced rats. The activities of superoxide dismutase, catalase and glutathione peroxidase decreased significantly in the erythrocytes and tissues of hypertensive rats and treatment with sesamol resulted in a marked increase in the activities of enzymatic antioxidants.

**Table 1:** Effect of sesamol on TBARS, LOOH and CD in plasma and tissues of UNX-control and DOCA-salt hypertensive rats

Groups		UNX-control	UNX-control + sesamol (200 mg/kg BW)	DOCA-salt	DOCA-salt + sesamol (50 mg/kg BW)	DOCA-salt + sesamol (100 mg/kg BW)	DOCA-salt + sesamol (200 mg/kg BW)
TBARS	Plasma (mmol/dL)	0.16 ± 0.01^a^	0.27 ± 0.02^b^	0.43 ± 0.04^c^	0.18 ± 0.01^d^	0.26 ± 0.01^b^	0.38 ± 0.02^e^
	Liver (mmol/100 g wet tissue)	0.84 ± 0.08^a^	1.95 ± 0.13^b^	2.42 ± 0.13^c^	0.92 ± 0.05^a^	1.37 ± 0.05^d^	2.12 ± 0.05^e^
	Kidney (mmol/100 g wet tissue)	1.46 ± 0.05^a^	2.05 ± 0.12^b^	3.94 ± 0.27^c^	1.59 ± 0.08^a^	1.87 ± 0.05^d^	2.75 ± 0.20^e^
	Heart (mmol/100 g wet tissue)	0.57 ± 0.03^a^	1.99 ± 0.04^b^	3.18 ± 0.06^c^	0.66 ± 0.04^d^	1.95 ± 0.06^b^	2.60 ± 0.06^e^
LOOH	Plasma (mmol/dL)	9.00 ± 0.75^a^	14.82 ± 0.63^b^	19.76 ± 0.85^c^	10.65 ± 0.83^d^	13.67 ± 0.45^e^	17.77 ± 0.96^f^
	Liver (mmol/100 g wet tissue)	76.19 ± 5.83^a^	94.70 ± 6.00^b,e^	107.14 ± 9.35^c^	82.32 ± 5.99^d^	91.37 ± 9.96^b^	87.93 ± 6.6^e^
	Kidney(mmol/100 g wet tissue)	65.6 ± 1.98^a^	109.82 ± 4.62^b^	158.33 ± 7.60^c^	79.76 ± 4.32^d^	96.19 ± 4.94^e^	134.76 ± 5.28^f^
	Heart (mmol/100 g wet tissue)	65.18 ± 2.65^a^	91.61 ± 3.24^b^	130.36 ± 6.68^c^	75.95 ± 3.9^d^	83.27 ± 4.85^e^	103.57 ± 3.19^f^
CD	Plasma (mmol/dL)	0.66 ± 0.03^a^	0.93 ± 0.03^b^	1.13 ± 0.05^c^	0.72 ± 0.03^d^	0.85 ± 0.04^e^	0.99 ± 0.06^f^
	Liver(mmol/100 g wet tissue)	68.23 ± 1.73^a^	89.40 ± 1.84^b^	97.78 ± 1.97^c^	70.1 ± 4.81^a^	80.5 ± 4.65^d^	93.65 ± 1.86^e^
	Kidney (mmol/100 g wet tissue)	19.27 ± 1.79^a^	33.83 ± 2.33^b^	38.30 ± 2.54^c^	21.5 ± 1.47^d^	28.73 ± 2.13^e^	37.4 ± 2.89^c^
	Heart (mmol/100 g wet tissue)	40.08 ± 2.71^a^	57.27 ± 3.61^b^	70.32 ± 5.47^c^	45.78 ± 3.56^d^	56.63 ± 4.39^e^	63.47 ± 4.61^f^

Values are mean ± SD for six rats. Values not sharing a common superscript differ significantly at *p* < 0.05 (DMRT)

**Table 2:** Effect of sesamol on vitamin E, vitamin C and GSH in the plasma and tissues of UNX-control and DOCA-salt hypertensive rats

Groups		UNX-control	UNX-control + sesamol (200 mg/kg BW)	DOCA-salt	DOCA-salt + sesamol (50 mg/kg BW)	DOCA-salt + sesamol (100 mg/kg BW)	DOCA-salt + sesamol (200 mg/kg BW)
Vitamin E	Plasma (mg/dL)	1.84 ± 0.12^a^	1.22 ± 0.09^b^	0.90 ± 0.08^c^	1.71 ± 0.09^d^	1.35 ± 0.11^e^	1.14 ± 0.10^b^
	Liver (μg/mg protein)	6.01 ± 0.48^a^	4.84 ± 0.36^b,d^	3.45 ± 0.33^c^	5.84 ± 0.37^a^	5.07 ± 0.31^d^	4.51 ± 0.27^b^
	Kidney (μg/mg protein)	4.06 ± 0.32^a^	2.71 ± 0.14^b^	1.52 ± 0.13^c^	3.86 ± 0.16^a^	3.05 ± 0.22^d^	2.55 ± 0.15^b^
	Heart (μg/mg protein)	4.13 ± 0.22^a^	2.71 ± 0.12^b^	1.67 ± 0.11^c^	3.98 ± 0.23^a^	3.40 ± 0.28^d^	2.61 ± 0.22^b^
Vitamin C	Plasma (mg/dL)	2.05 ± 0.10^a^	1.66 ± 0.08^b^	0.95 ± 0.03^c^	1.91 ± 0.06^d^	1.78 ± 0.07^e^	1.48 ± 0.07^f^
	Liver (μg/mg protein)	0.74 ± 0.04^a^	0.55 ± 0.04^b,e^	0.49 ± 0.02^c^	0.69 ± 0.05^d^	0.58 ± 0.04^e^	0.53 ± 0.03^b^
	Kidney (μg/mg protein)	0.68 ± 0.04^a^	0.47 ± 0.30^b^	0.34 ± 0.02^c^	0.60 ± 0.04^d^	0.53 ± 0.03^e^	0.41 ± 0.02^f^
	Heart (μg/mg protein)	0.61 ± 0.03^a^	0.45 ± 0.02^b^	0.25 ± 0.02^c^	0.51 ± 0.04^d^	0.47 ± 0.02^b^	0.37 ± 0.03^e^
GSH	Plasma (mg/dL)	34.31 ± 1.83^a^	25.24 ± 1.74^b^	20.53 ± 1.02^c^	32.53 ± 1.47^a^	27.55 ± 1.84^d^	23.11 ± 1.8b^e^
	Liver (μg/mg protein)	11.86 ± 1.05^a^	9.52 ± 0.81^b,e^	8.03 ± 0.63^c^	10.82 ± 0.98^d^	9.93 ± 0.83^e,d^	8.85 ± 0.82^b,c^
	Kidney (μg/mg protein)	10.14 ± 1.01^a^	8.15 ± 0.70^b^	5.07 ± 0.43^c^	9.14 ± 0.75^d^	8.44 ± 0.61^b,d^	7.15 ± 0.71^e^
	Heart (μg/mg protein)	8.08 ± 0.77^a^	6.38 ± 0.53^b,d^	4.49 ± 0.41^c^	7.66 ± 0.69^a^	7.72 ± 0.74^d^	5.89 ± 0.57^b^

Values are mean ± SD for six rats. Values not sharing a common superscript differ significantly at *p* < 0.05 (DMRT)

**Table 3:** Effect of sesamol on SOD, catalase and GPx in erythrocytes and tissues of UNX-control and DOCA-salt hypertensive rats

Groups		UNX-control	UNX-control + sesamol (200 mg/kg BW)	DOCA-salt	DOCA-salt + sesamol (50 mg/kg BW)	DOCA-salt + sesamol (100 mg/kg BW)	DOCA-salt + sesamol (200 mg/kg BW)
SOD	Erythrocyte (IU^A^/mg Hb)	7.39 ± 0.62^a^	5.72 ± 0.51^b,e^	3.25 ± 0.32^c^	6.76 ± 0.65^d^	6.01 ± 0.60^e^	5.13 ± 0.46^b^
	Liver (IU^A^/mg protein)	8.10 ± 0.44^a^	6.03 ± 0.33^b^	4.37 ± 0.24^c^	7.08 ± 0.41^d^	6.57 ± 0.35^e^	5.87 ± 0.45^b^
	Kidney (IU^A^/mg protein)	14.90 ± 1.09^a^	11.24 ± 0.63^b^	8.93 ± 0.34^c^	13.48 ± 1.03^d^	12.63 ± 0.61^d^	10.16 ± 0.56^e^
	Heart (IU^A^/mg protein)	5.38 ± 0.36^a^	4.07 ± 0.19^b^	3.10 ± 0.25^c^	4.93 ± 0.42^d^	4.48 ± 0.27^e^	3.80 ± 0.21^b^
CAT	Erythrocyte (IU^B^/mg Hb)	165.61 ± 4.07ª	134.03 ± 3.93^b^	94.39 ± 3.30^c^	160.30 ± 4.14^d^	147.62 ± 2.64^e^	125.55 ± 3.04^f^
	Liver (IU^B^/mg protein)	75.08 ± 6.82^a^	65.43 ± 3.20^b^	53.61 ± 4.00^c^	73.14 ± 4.45^a^	67.44 ± 3.52^b^	59.93 ± 2.47^e^
	Kidney (IU^B^/mg protein)	30.90 ± 2.71^a^	22.25 ± 1.41^b^	17.18 ± 1.17^c^	28.14 ± 2.06^d^	24.96 ± 1.39^e^	20.19 ± 1.40^b^
	Heart (IU^B^/mg protein)	51.79 ± 4.70^a^	38.64 ± 1.84^b^	25.13 ± 1.02^c^	49.85 ± 3.19^ª^	42.91 ± 2.49^d^	32.71 ± 2.96^e^
GPx	Erythrocyte (IU^C^/mg Hb)	15.11 ± 1.55^a^	9.86 ± 0.84^b^	6.23 ± 0.57^c^	14.18 ± 1.38^a^	11.93 ± 0.97^d^	8.82 ± 0.85^b^
	Liver (IU^C^/mg protein)	7.88 ± 0.70^a^	5.93 ± 0.42^b^	4.86 ± 0.48^c^	7.30 ± 0.64^d,a^	6.81 ± 0.58^d^	5.09 ± 0.51^b^
	Kidney (IU^C^/mg protein)	8.82 ± 0.85^a^	5.53 ± 0.43^b^	3.20 ± 0.30^c^	7.73 ± 0.69^d^	6.47 ± 0.44^e^	4.72 ± 0.40^f^
	Heart (IU^C^/mg protein)	7.62 ± 0.58^a^	4.79 ± 0.40^b^	3.17 ± 0.21^c^	6.30 ± 0.42^d^	5.32 ± 0.48^b^	4.07 ± 0.30^e^

Values are mean ± SD for six rats. Values not sharing a common superscript letters differ significantly at *p* < 0.05 (DMRT)

^A^Enzyme concentration required to inhibit the NBT to 50 % in 1 min

^B^μmol of H_2_O_2_ consumed per minute

^C^μg of GSH utilized per minute

**Fig. 4:**
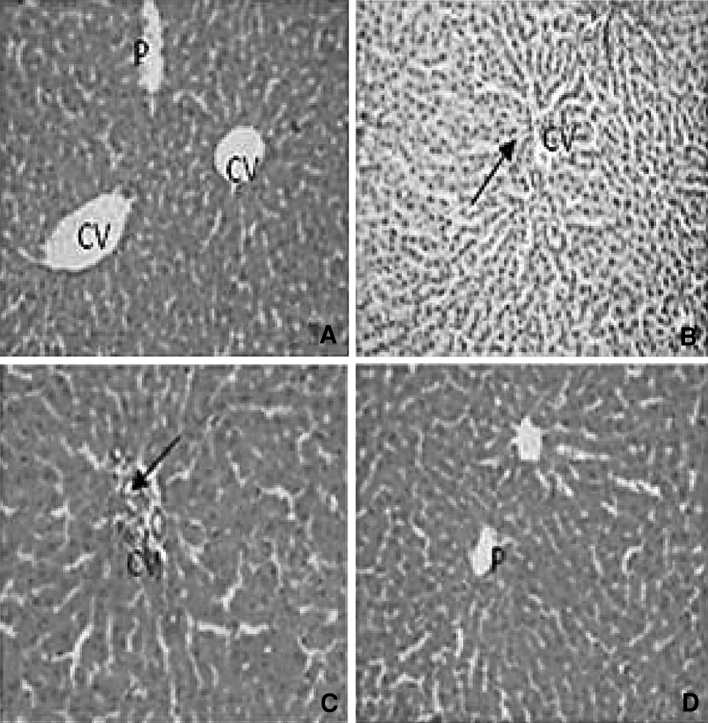
Effect of sesamol on liver morphology (H&E X40) in hypertensive rats. **a** UNX-control rat showing normal central vein (*CV*) and portal tract (*P*). **b** and **c** reveal liver section of DOCA-salt rat. **b** hepatocytes with foamy degeneration, dilated and congested central vein (*right arrow*) and **c** periportal fibrosis (*right arrow*) and loss of cell boundaries. **d** DOCA-salt rats with sesamol showing mild thickened arteriole with inflammatory cells and portal triad (*P*) showing normal morphology

Histopathological evaluation of liver in UNX-control group showed normal morphology of the central vein (Fig. 4a). Morphological changes including hepatocytes with foamy degeneration, dilated congested central vein and periportal fibrosis and inflammation were observed in the DOCA-salt-induced hypertensive rats (Fig. 4b, c). Sesamol-treated hypertensive rats showed a mild thickened arteriole with inflammatory cells and portal triad (Fig. 4d).

**Fig. 5:**
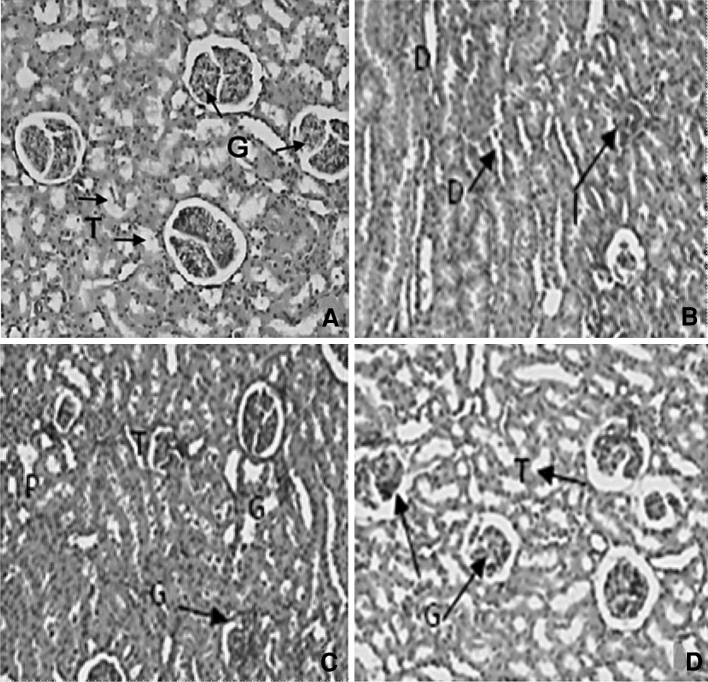
Effect of sesamol on kidney morphology (H&E X40) in hypertensive rats. **a** UNX-control rat showing normal glomeruli (*G*) and tubules (*T*). **b** and **c** reveal kidney section of DOCA-salt rat. **b** dilated lumen of distal convoluted tubules (*D*), protein precipitation (*P*) in tubules and cloudy swelling and **c** arteriolar thickening and inflammatory cell infiltration (*I*). **d** DOCA-salt rats with sesamol showing mild tubular dilation and normal glomeruli

Figure 5 illustrates the histopathological changes of kidney tissues of UNX-control and DOCA-salt hypertensive rats. UNX rats showed normal glomeruli and tubules (Fig. 5a). DOCA-salt hypertensive rats’ kidney revealed the dilated lumen of distal convoluted tubules, protein precipitation in tubules and cloudy swelling (Fig. 5b), and arteriolar thickening and inflammatory cell infiltration (Fig. 5c). Administration of sesamol to DOCA-salt hypertensive remarkably reduced the above changes, showing mild tubular dilation and normal glomeruli (Fig. 5d).

**Fig. 6:**
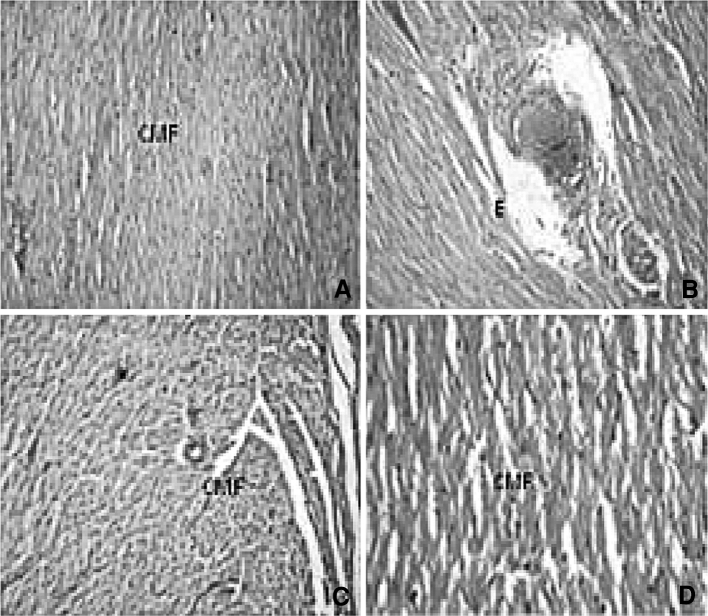
Effect of sesamol on heart morphology (H&E X40) in hypertensive rats. **a** UNX-control rat showing normal cardiac muscle fibres (*CMF*). **b** and **c** illustrate heart section of DOCA-salt rat. **b** coronary vessel with oedema (*E*) of wall and thickening of coronary vessel wall, and **c** shows cardiac muscle fibre rupture and mononuclear infiltration. **d** DOCA-salt rats with sesamol showing mild inflammation and normal cardiac fibres

Histopathological changes of the heart in UNX-control and DOCA-salt-induced hypertensive rats are shown in Fig. 6. UNX-control rats showed normal cardiac muscle fibre (Fig. 6a). Hypertensive rats showed a coronary vessel with oedema of wall and thickening of coronary vessel wall (Fig. 6b); Fig. 6c showed cardiac muscle fibre rupture and mononuclear infiltration, and treatment with sesamol at 50 mg/kg body weight brought these changes to near-normal architecture (Fig. 6d).

## Discussion

The prolonged administration of DOCA with salt drinking water in UNX rats has been reported to cause hypertension. The DOCA-salt rat, a non-genetic model, is an appropriate model for testing the effect of natural and synthetic compounds on cardiovascular remodelling and offers the possibility for the development of newer therapeutic agents [25]. DOCA-salt-administered rats absorb sodium (Na^+^) and water in the kidney, which raises circulating blood volume and produces the well-known volume-dependent hypertension. Earlier reports declared that increased vascular superoxide production and imbalance in antioxidant status coupled with an increase in lipid peroxidation occur in DOCA-salt hypertension. Increased aldosterone concentrations may activate oxidative stress through an unregulated NADPH oxidase in the DOCA-salt model [26].

The DOCA-salt-induced hypertensive rats showed elevated blood pressure as compared to the UNX rats. Amongst the three doses, the 50-mg dose remarkably reduced the blood pressure compared to the other higher doses. Sesamol might have reduced the vascular O_2_^−^ production by inhibiting the activity of NADPH oxidase, an enzyme which is known to be a main source of O_2_^−^ production [11] and is increased in the vascular tissues of DOCA-salt hypertensive rats. In addition, there was a positive correlation between blood pressure and vascular O_2_^−^ production, thereby indicating a close relationship between sesamol-induced decrease in vascular O_2_^−^ production and its antihypertensive effects. Higher concentrations of sesamol might have resulted in the production of many by-products that would have interfered with the activity. This can be inferred from our study, which demonstrated that 100- and 200-mg doses did not show the restoration of normal blood pressure.

Disruption of liver tissue architecture under hypertension is an indication of hepatocellular injury. Clinical diagnosis of disease and damage to the structural integrity of the liver is commonly assessed by monitoring the status of serum AST, ALP and ALT activities, which are sensitive serological indicators of liver toxicity. ALP and GGT are the membrane-bound enzymes and their elevation indicates membrane disruption in the organ. Increased AST and ALT release from damaged tissues has become a definitive diagnostic and prognostic criterion for various diseases and disorders. The increased activities of AST, ALT, ALP and GGT in serum might be mainly due to the leakage of these enzymes from the liver cells into the blood stream [27], which indicated the hepatotoxic effect of DOCA-salt. The reduced antioxidant status might also be involved in the hepatic injury because reactive free radicals are implicated as the potential mediators of tissue injury. In this study, the activities of these enzymes were found to increase in the group where hypertension was induced using DOCA-salt, but were significantly reduced in groups that received sesamol.

Free radical-mediated cell membrane lipid peroxidation has been implicated in pathological conditions such as increased cell membrane rigidity, decreased cellular deformability and lipid fluidity. Oxidative stress has been implicated in the pathogenesis of hypertension, which is responsible for higher levels of lipid peroxidative markers in hypertensive rats. The level of tissue damage has been evaluated by the measurement of lipid peroxidation products and antioxidants [28]. It has been reported that the products of lipid peroxidation disperse from the site of tissue damage and therefore can be measured in plasma [29]. An increased concentration of end products of lipid peroxidation is the evidence most frequently quoted for the involvement of toxic radicals in some diseases including hypertension.

In this study, the increase in the levels of TBARS, LOOH and CD in the plasma and tissues of DOCA-salt hypertensive rats might be due to insufficient antioxidant potential. After administration of sesamol, lipid peroxidation products decreased. Sesamol has been shown to significantly reduce lipid oxidation in the bovine and porcine model system [8]. The anti-lipid peroxidative effect of sesamol in DOCA-salt hypertensive rats might be due to diminished formation of hydroxyl radical and superoxide anion. Structurally, sesamol has two functional groups (phenolic ring and benzodioxyl group) capable of scavenging ROS. The benzodioxyl group scavenges hydroxyl radicals, which in surplus slow oxidative chain reactions such as the Harber–Weiss and Fenton reactions [30].

Antioxidants play a crucial role in scavenging ROS and protect the cells from oxidative damage. SOD is an extremely effective antioxidant enzyme and is responsible for catalytic dismutation of highly reactive and potentially toxic superoxide radicals to H_2_O_2_ [31]. CAT traps the harmful hydrogen peroxide and converts into water and oxygen. GPx, an oxidative stress-inducible enzyme, catalyses the reduction of hydrogen peroxide and hydroperoxide to non-toxic products and scavenges the highly reactive lipid peroxides in the aqueous phase of cell membrane [32]. GPx detoxifies peroxides with GSH, GSH acting as an electron donor in the reduction reaction, producing GSSG as an end product [33]. The enzymatic antioxidants exist in all oxygen-metabolizing cells, which protect cell and tissue damage from enhanced lipid peroxidation via elimination of ROS and provide a defence mechanism for oxidized membrane components. The inhibition of these enzymatic antioxidant activities is found to be involved in many degenerative diseases. Our results indicate that DOCA-salt-induced hypertension disrupts the actions of antioxidants enzymes. The decreased activities of these enzymes may be due to the production of ROS such as superoxide anion, hydrogen peroxide and hydroxyl radicals that reduce the activity of these enzymes. Sesamol administration significantly restored the activities of these enzymes in erythrocyte and tissues of DOCA-salt rats, and it was also reported that sesamol possesses superoxide-scavenging and powerful antioxidant activities during disease conditions [34]. The overexpression of SOD might be an adaptive response and it results in increased dismutation of superoxide to hydrogen peroxide. The rise in the activity of GPx could be due to its induction to counter the effect of increased oxidative stress. Phenolics are powerful hydrogen-donating antioxidants and free radical scavengers in several in vitro and in vivo models. Studies on the antioxidant potency of various phenolics have confirmed the importance of the distribution and quantity of the hydroxyl groups. Hsu et al. [35] have reported that the hydroxyl group of sesamol increased SOD, CAT and GPX activities in endotoxin-induced oxidative stress and multiple organ injury rats.

Non-enzymatic antioxidants such as reduced glutathione, vitamin C and vitamin E play an excellent role in protecting the cells from oxidative damage. Vitamin functions as a free radical scavenger of O_2_^•−^ radicals and successfully prevents detectable oxidative damage under all types of oxidative stress. Vitamin E, a membrane stabilizer, is an important nutrient which functions as a chain-breaking antioxidant which prevents the propagation of free radical reactions in all cell membranes in the human body [36]. Vitamin C, a hydrophilic antioxidant, appears to trap the peroxyl radical in the aqueous phase with a rate large enough to interrupt virtually all these radicals before they can diffuse into plasma lipids [37]. GSH, one of the most abundant non-enzymatic antioxidant biomolecules, is considered to be a free radical scavenger by the role of a sulfhydryl (SH) group provider or a cofactor for protective enzymes, which plays a pivotal role in the cellular defence against oxidative damage [38]. A consistent finding concerning the GSH cycle alterations in hypertension is decreased GPx activity, described in other hypertension experimental models. A reduced GPx activity leads to increased cellular accumulation of ROS and lipid peroxidation. Decreased GSH concentration may also contribute to decreased GPx activity because GSH is one of the substrates for GPx [39].

In our study, a significant decrease in the levels of plasma and tissue GSH, ascorbic acid and vitamin E in DOCA-salt-treated rats was observed. The decrease in vitamin E, in hypertensive rats, might be due to the increased utilization in scavenging the oxyradicals generated or due to decreased vitamin C level because there is a well-established relation between vitamin E and vitamin C. The decrease in the levels of these non-enzymatic antioxidant parameters may be due to the increased turnover for preventing oxidative damage in these animals, suggesting an increased defence against oxidant damage in hypertension. In sesamol-administered rats, the level of vitamin E increased, which might be due to decreased lipid peroxidation. The antioxidant activity of phenolics is mainly due to their redox properties, which allow them to act as reducing agents, hydrogen donators and singlet oxygen quenchers. In addition, sesamol molecules have a metal chelation potential [40].

The DOCA-salt-induced rats showed hepatocytes with foamy degeneration, dilated congested central vein and periportal fibrosis, and loss of cell boundaries. DOCA-salt hypertensive rats’ kidney revealed the dilated lumen of distal convoluted tubules, protein precipitation in tubules and cloudy swelling, and arteriolar thickening and inflammatory cell infiltration. Hypertensive rats showed cardiac muscle fibre rupture and mononuclear infiltration. Administration of sesamol at a 50-mg dose brought the above-mentioned changes in liver, kidney and heart to near-normal architecture.

Thus, administration of sesamol at 50 mg/kg BW to hypertensive rats significantly reduced blood pressure and improved the antioxidant defence mechanism. The histopathological studies also supported the biochemical findings.
